# Supplementary material for: Fish-hunting cone snail venoms are a rich source of minimized ligands of the vertebrate insulin receptor
Source: eLife. 2019 Feb 12;8:e41574. doi: 10.7554/eLife.41574 (PMC6372279; doi:10.7554/eLife.41574)
Supplement: Transparent reporting form [file elife-41574-transrepform.pdf]

eLIFE
1st Floor
24 Hills Road
Cambridge CB2 1JP, UK
P 01223 855340
W elifesciences.org
T @elife

# *eLife's* transparent reporting form

We encourage authors to provide detailed information *within their submission* to facilitate the interpretation and replication of experiments. Authors can upload supporting documentation to indicate the use of appropriate reporting guidelines for health-related research (see EQUATOR Network), life science research (see the BioSharing Information Resource), or the ARRIVE guidelines for reporting work involving animal research. Where applicable, authors should refer to any relevant reporting standards documents in this form.

If you have any questions, please consult our Journal Policies and/or contact us: editorial@elifesciences.org.

**Sample-size estimation**

- You should state whether an appropriate sample size was computed when the study was being designed
- You should state the statistical method of sample size computation and any required assumptions
- If no explicit power analysis was used, you should describe how you decided what sample (replicate) size (number) to use

Please outline where this information can be found within the submission (e.g., sections or figure legends), or explain why this information doesn't apply to your submission:

Information on sample size is provided for all experiments in
lines 165-170 for data shown in Figure 2;
line 195-196 for Figure 3;
line 213 for Figure 4;
and line 246 for Figure 5.
Sample size was selected by weighing the expense of data collection and the need to have sufficient statistical power. This differed for each experiment and was of particular importance for in vivo experiments where ethical concerns were weighed in. All in vivo studies were carefully revised and approved by the University of Utah Institutional Animal Care and Use Committee.
More information on sample size selection is provide below.
For data shown in Figure 2 (zebrafish model of diabetes) each data point represents an individual fish since repeated blood draws are not feasible and not permitted by our Institutional Animal Care and Use facility. Thus, we limited the number of animals in this assay to those needed to first establish the model and then show that venom insulins significantly lowered blood glucose compared to non-treated fish. However, we decided not to determine significant difference between the different venom insulins since this was not the main focus of this study and would have required a very large number of fish.
For data shown in Figure 3 we again wanted to establish that venom insulins are indeed capable of binding to the insulin receptor which was successfully determined by using 2 biological replicates with 3 technical replicates each.
For data shown in Figure 4 we wanted to establish that receptor binding leads to downstream insulin signaling which was successfully shown with the sample size used. Rather than comparing the activities of each compound (which would have required a larger sample size) we decided to categorize venom insulins into two main categories: those that are (1) more or (2) less active than the B-chain truncated analog of human insulin, DOI.
For data shown in Figure 5 we were more careful with sample size selection because these experiments required animal use. In order to establish this in vivo mouse model we initially used 5 animals for the control group (human insulin) but then only used 3 mice for each of the different venom insulins to keep the sample size small (while still obtaining meaningful data). We also decided not to test every single venom insulin but only selected the three insulins that showed highest in vitro activity in each of the three species of cone snail. Data collected was sufficient to show that these venom insulins were potent in a mammalian model of diabetes and that their in vivo activities correlated with in vitro insulin receptor activation data.

**Replicates**

- You should report how often each experiment was performed
- You should include a definition of biological versus technical replication
- The data obtained should be provided and sufficient information should be provided to indicate the number of independent biological and/or technical replicates
- If you encountered any outliers, you should describe how these were handled

- Criteria for exclusion/inclusion of data should be clearly stated
- High-throughput sequence data should be uploaded before submission, with a private link for reviewers provided (these are available from both GEO and ArrayExpress)

Please outline where this information can be found within the submission (e.g., sections or figure legends), or explain why this information doesn't apply to your submission:

Information on sample size is provided for all experiments in
lines 165-170 for data shown in Figure 2;
line 195-196 for Figure 3;
line 213 for Figure 4;
and line 246 for Figure 5.
Data is provided in Supporting Source File ("Source.Files.Figures2-5")

**Statistical reporting**

- Statistical analysis methods should be described and justified
- Raw data should be presented in figures whenever informative to do so (typically when N per group is less than 10)
- For each experiment, you should identify the statistical tests used, exact values of N, definitions of center, methods of multiple test correction, and dispersion and precision measures (e.g., mean, median, SD, SEM, confidence intervals; and, for the major substantive results, a measure of effect size (e.g., Pearson's r, Cohen's d)
- Report exact p-values wherever possible alongside the summary statistics and 95% confidence intervals. These should be reported for all key questions and not only when the p-value is less than 0.05.

Please outline where this information can be found within the submission (e.g., sections or figure legends), or explain why this information doesn't apply to your submission:

Information is provided in
lines 177-181 for data shown in Figure 2;
lines 195-200 for Figure 3 (figure caption) and in right panel of Figure;
lines 231-232 (figure caption) and panel E for Figure 4;
lines 270-271 for Figure 5.

(For large datasets, or papers with a very large number of statistical tests, you may upload a single table file with tests, Ns, etc., with reference to sections in the manuscript.)

**Group allocation**

- Indicate how samples were allocated into experimental groups (in the case of clinical studies, please specify allocation to treatment method); if randomization was used, please also state if restricted randomization was applied
- Indicate if masking was used during group allocation, data collection and/or data analysis

Please outline where this information can be found within the submission (e.g., sections or figure legends), or explain why this information doesn't apply to your submission:

Does not apply to this manuscript

**Additional data files ("source data")**

- We encourage you to upload relevant additional data files, such as numerical data that are represented as a graph in a figure, or as a summary table
- Where provided, these should be in the most useful format, and they can be uploaded as "Source data" files linked to a main figure or table
- Include model definition files including the full list of parameters used
- Include code used for data analysis (e.g., R, MatLab)
- Avoid stating that data files are "available upon request"

Please indicate the figures or tables for which source data files have been provided:

Data is provided in Supporting Source File ("Source.Files.Figures2-5")
Sequences of insulins identified and characterized in this study are shown in Figure 1 (mature sequences), Supporting Figure 1 (full-length sequences) and have been deposited in the NCBI Protein Database (Accession Numbers: Con-Ins G1: AJD85832; Con-Ins G3: AJD85820; Con-Ins T1A: KP268600; Con-Ins T1B: KP268611; Con-Ins T2: MH879035; Con-Ins K1: MH879033; Con-Ins K2: MH87903; ,release date: December 1, 2018).
Coordinates for Molecular Dynamics Simulations shown in Figure 6 are provided as pdb files ("Source File Fig. 6, 1-3").
